# Dual immunological and proliferative regulation of immune checkpoint FGL1 in lung adenocarcinoma: The pivotal role of the YY1–FGL1–MYH9 axis

**DOI:** 10.3389/fimmu.2022.1014053

**Published:** 2022-10-04

**Authors:** Xi-Yang Tang, Yan-Lu Xiong, Ya-Bo Zhao, Jie Yang, An-Ping Shi, Kai-Fu Zheng, Yu-Jian Liu, Chen Shu, Tao Jiang, Nan Ma, Jin-Bo Zhao

**Affiliations:** ^1^ Department of Thoracic Surgery, Tangdu Hospital, Air Force Medical University, Xi’an, China; ^2^ Department of Radiology, Functional and Molecular Imaging Key Lab of Shaanxi Province, Tangdu Hospital, Fourth Military Medical University (Air Force Medical University), Xi’an, China; ^3^ Department of Ophthalmology, Tangdu Hospital, Air Force Medical University, Xi’an, China

**Keywords:** immunity, proliferation, FGL1, YY1, MYH9

## Abstract

**Rational:**

Lung cancer is the most common tumor worldwide, with the highest mortality rate and second highest incidence. Immunotherapy is one of the most important treatments for lung adenocarcinoma (LUAD); however, it has relatively low response rate and high incidence of adverse events. Herein, we explored the therapeutic potential of fibrinogen-like protein 1 (FGL1) for LUAD.

**Methods:**

Data from GEPIA and ACLBI databases were assessed to explore gene–gene correlations and tumor immune infiltration patterns. A total of 200 patients with LUAD were recruited. FGL1 levels in the serum and cellular supernatant were determined by enzyme-linked immunosorbent assay. *In vitro* and *in vivo* experiments were performed to assess the effect FGL1 on the proliferation of LUAD cells. Cocultures were performed to explore the effect of FGL1 knockdown in lung cancer cells on T cells, concerning cytokine secretion and viability. PROMO and hTFtarget databases were used for transcription factor prediction. Quantitative polymerase chain reaction (qPCR), chromatin immunoprecipitation, and dual luciferase reporter assays were performed to validate the identified transcription factor of FGL1. Immunoprecipitation, mass spectrometry and gene ontology analysis were performed to explore the downstream partners of FGL1.

**Results:**

FGL1 expression in LUAD was positively associated with *PDL1*, but not for *PD1* expression. Moreover, FGL1 was positively associated with the *CD3D* expression and negatively associated with *FOXP3*, *S100A9*, and *TPSB2* within the tumor site. FGL1 promotes the secretion of interleukin-2 by T cells *in vitro*, simultaneously inducing their apoptosis. Indeed, YY1 is the upstream molecule of FGL1 was found to be transcriptionally regulated by YY1 and to directly by to *MYH9* to promote the proliferation of LUAD cells *in vitro* and *in vivo*.

**Conclusions:**

FGL1 is involved in the immunological and proliferative regulation of LUAD cells by controlling the secretion of important immune-related cytokines *via* the YY1–FGL1–MYH9 axis. Hence, targeting FGL1 in LUAD may pave the way for the development of new immunotherapies for tackling this malignancy.

## Graphical Abstract

FGL1 may promote the secretion of IL-2, inducing the apoptosis of T cells. The YY1-FGL1-MYH9 axis is active in the FGL1-mediated regulation of proliferation, and is possibly involved in cytokine secretion.

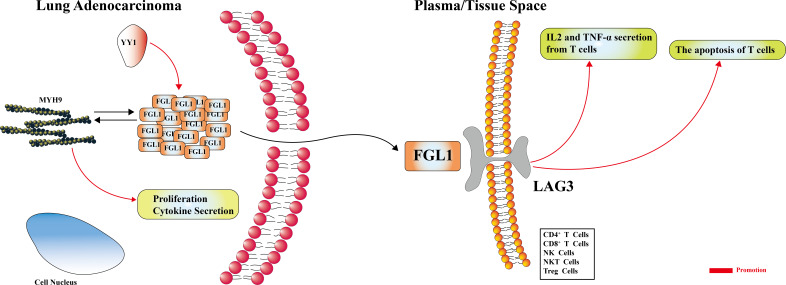

## Introduction

Lung cancer is the most common malignancy worldwide, with lung adenocarcinoma (LUAD) being the most common cancer type in both men and women. Lung cancer has the highest fatality rate (21% in males and females) and the second highest incidence (12% in males and 13% in females) among all tumors, even for distant metastasis lung cancer, with an average 5-year survival rate of 6% (1). Currently, immunotherapy is one of the most important treatment approaches used to tackle these cancers with good therapeutic effect. Nonetheless, some intractable problems remain unsolved; for example, the response rate of anti-PD1/PD-L1 treatment is still low, only reaching approximately 30%, in non-small cell lung cancer (2). Moreover, meta-analysis data comprising 22 clinical trials showed that the immune-related adverse events incidence of anti-CTLA-4 therapy reached up to 70% (3). Therefore, improved immunotherapies with higher response rate and fewer adverse effects are urgently needed.

Recent evidence suggests that the fibrinogen-like protein 1 (FGL1), also known as hepassocin, HRFREP-1, and FREP1 (4–7), may be a valuable anticancer therapeutic target. FGL1 is a ligand of the lymphocyte-activation gene 3 (LAG3), which is mainly expressed on the surface and cytoplasm of lung and breast cancer cells, and is believed to prevent the activation of T cells (8, 9). Yin Yang 1 (YY1) is a 45 KDa multifunctional transcription factor that is involved in cellular and viral gene expression. The direct and indirect activation, as well as repression of YY1 occurs through cofactor recruitment or the disruption of its binding sites (10). Myosin heavy chain 9 (MYH9) is a 230 KDa cytoskeletal protein that promotes cell motility by interacting with RAB3A (11), ZBED4 (12) and S100A4 (13). In particular, various reports demonstrated that MYH9 is involved in tumor proliferation, migration, and metastasis (14–16), in this study, YY1 and MYH9 may be the upstream and downstream molecule of FGL1, respectively. To date, most investigations explored how FGL1 can promote the activation and infiltration of T cells in LUAD (17), whereas its other potential roles remain unclear. Moreover, FGL1 was proven to be expressed at higher levels in LUAD than in paracancerous tissues and was shown to regulate the proliferation of LUAD cells (18), the underlying molecular mechanisms involved in this process were not elucidated. Herein, we demonstrate for the first time that FGL1 can induce T cell apoptosis and that FGL1-mediated immunity and proliferation regulation is dependent on the YY1-FGL1-MYH9. These finding provide new evidences that suggest that FGL1 may represent a valuable target for the development of novel anti-cancer immunotherapies.

## Methods

### Analysis of gene expression and immune infiltration

Data from the Gene Expression Profiling Interactive Analysis (GEPIA) database (http://gepia.cancer-pku.cn/), which comprises RNA-sequencing results from the Genotypic-Tissue Expression project and The Cancer Genome Atlas, were used for gene expression and gene-gene correlation analyses (19). Data from the Assistant for Clinical Bioinformatics (ACLBI) database (www.aclbi.com) were used to perform immune infiltration analysis.

### Evaluation of transcription factors

The Human Transcription Factors Target (hTFtarget) database (https://bio.tools/hTFtarget), which comprises information on 659 transcription factors obtained from 7,190 experimental samples (20), and the PROMO database (http://alggen.lsi.upc.es/cgi-bin/promo_v3/promo/promoinit.cgi?dirDB=TF_8.3), which helps to identify putative transcription factor binding sites in DNA sequences (21), were used to predict potentially valuable transcription factors.

### LUAD sample and healthy donor serum collection

A total of 200 LUAD intratumoral samples, as well as 200 peritumoral samples, were collected from patients who were surgically treated in Tangdu Hospital. The inclusion criteria for this study were the following: i) pathological diagnosis of LUAD; ii) pre-operatively laboratory and imaging examinations available; and iii) complete and available follow-up data. Exclusion criteria were the following: i) chemotherapy, radiotherapy, or targeted therapy performed before surgery; ii) serious lung-, heart-, brain-, and other important organ-related diseases; and/or iii) incomplete follow-up data or lack of laboratory and imaging results. In addition, 12 serum samples from four healthy donors were collected, with 32 serum samples from 16 LUAD patients, and these were used for ELISA to detect the level of FGL1. The study was conducted in completed accordance with the Declaration of Helsinki (as revised in 2013) and all participants provided written informed consent. This study was proved by Ethics Committee of the Air Force Medical University (No. 202003-018).

### Immunohistochemical staining

All tissue samples were embedded in paraffin and cut into 3-μm slices. Next, they were dewaxed with serial 75% and 85% alcohol solutions, followed by washes with absolute ethyl alcohol and Xylene I/II/III solutions. The samples were then placed in autoclaved citric acid buffer (pH 6.0) and boiled for 15 min, and then on 3% hydrogen peroxide and incubated for 20 min. The tissue slices were then coated overnight at 4 °C with anti-FGL1 polyclonal antibody (1:100 dilution; Proteintech, Rosemont, IL, USA). The samples were washed three times times with phosphate buffer saline. Next, a secondary antibody conjugated with horseradish peroxidase (1:200 dilution; Servicebio, Wuhan, China) was incubated at 25 °C for 50 min. The samples were washed three times and 3,3-diaminobenzidine (Servicebio) was used to detect positive antibody binding. The samples were counterstained with hematoxylin.

### Enzyme-linked immunosorbent assay (ELISA)

Human FGL1 levels were evaluated using commercially available ELISA kits (Cloud-Clone Corp. Wuhan, China), and human interferon (IFN)-γ (EK180-96), tumor necrosis factor (TNF)-α (EK182-96), and interleukin (IL)-2 (EK102-96) levels were evaluated using commercially available ELISA kits (all from Multisciences, Hangzhou, China), according to the manufacturer’s protocols.

### Cell culture

PC9 and HCC827 human lung cancer cells and HBE human bronchial epithelial cells (iCell Bioscience Inc., Shanghai, China) were cultured at 37 °C and 5% CO_2_ atmosphere, and seeded in Gibco RPMI 1640 medium (Thermo Fisher Scientific, Waltham, MA, USA) supplemented with 10% fetal calf serum (FCS, Thermo Fisher Scientific, Waltham, MA, USA); the cellular supernatant was collected for ELISA. Lewis lung carcinoma cells (human; iCell Bioscience Inc.) were cultured at 37 °C and 5% CO_2_ atmosphere, and seeded in Gibco Dulbecco’s Modified Eagle Medium (DMEM; Thermo Fisher Scientific) supplemented with 10% FCS. 293T cells (human; Beijing TsingKe Biotech Co., Beijing, China) were cultured at 37 °C and 5% CO_2_ atmosphere, and seeded in Gibco DMEM (Thermo Fisher Scientific) supplemented with 10% FCS. Jurkat T cells (human; iCell Bioscience Inc.) were cultured at 37 °C and 5% CO_2_ atmosphere, and seeded in Gibco RPMI 1640 (Thermo Fisher Scientific) supplemented with 12% FCS.

### *FGL1* knockdown and overexpression

FGL1 was knocked-down (KD) in PC9, HCC827, and Lewis lung carcinoma cells using pHBLV-U6-FGL1-shRNA-EF1a-EGFP-T2A-PURO vector synthesized by HanBio Therapeutics (Shanghai, China). The sequences to target *FGL1* were 5’–GGAGGAGGATGGACTGTAA–3’ and 5’–TTACAGTCCATCCTCCTCC–3’. The stable knockdown efficiency using this approach was demonstrated elsewhere (18). FGL1 was overexpressed (OE) in PC9 and HCC827 cells using the HBLV-h-FGL1-3xflag-PURO vector synthesized by HanBio Therapeutics (Shanghai, China). The stable FGL1 overexpression sequence can be found in the raw data, and the efficiency using this approach was demonstrated by western blotting.

### *YY1* knockdown

YY1 was knocked-down in PC9 and HCC827 cells by small interfering RNA (siRNA), which was synthesized by RiboBio (Guangzhou, China), the sequence was as follow: 5’- GATGGTTGTAATAAGAAGT -3’.

### Cell coculture

Direct coculture was performed in 10-cm culture dishes. Briefly, FGL1_NC (negative control) and FGL1_KD PC9 cells (5×10^5^ cells) were seeded and cultured in RPMI 1640 until the cell density achieved 70%. Jurkat cells were activated for 48 h using phytohemagglutinin (HY-N7038; MedChemExpress, Monmouth Junction, NJ, USA); then, were cocultured (3×10^6^ activated cells) directly with the PC9 cells for 48 h. The cocultured Jurkat cells were collected for apoptosis analysis and the cellular supernatant was collected for ELISA analysis.

### Flow cytometry

Jurkat cells cocultured with FGL1_NC and FGL1_KD PC9 cells were collected, washed once with phosphate buffer saline and then with (1×) binding buffer, and resuspended in 100 μL (1×) binding buffer. Fluorochrome-conjugated Annexin V (5 µL) was added to the solution and incubated at room temperature for 10–15 min protected from light. The cells were washed with (1×) binding buffer, resuspend in 200 μL binding buffer, 5 µL of Propidium Iodide Staining Solution was added, and were immediately analyzed (within 4 h) by flow cytometry (Beckman Coulter, Brea, CA, USA).

### Western blotting

Total protein collection kit (Invent Biotechnologies Inc., Plymouth, MN, USA) was used for total protein collection. Protein loading buffer (5×) was added to the collected protein samples, which were then boiled for 10 min. The concentration of the extracted protein samples was estimated using bovine serum albumin as control (Beyotime, Shanghai, China). The protein samples were then separated using 10% sodium dodecyl sulfate-polyacrylamide gels and electrophoretically (300 mA for 60 min) transferred onto nitrocellulose membranes. QuickBlock (Beyotime) was used to block the membranes at room temperature for 15 min. the membranes were incubated overnight at 4 °C with Anti-GADPH antibody (1:10000 dilution; Catalog: ab181602, abcam, UK), anti-β-actin antibody (1:20000 dilution; Catalog: 66009-1-Ig, Proteintech, China), anti-FGL1 polyclonal antibody (1:250 dilution; Catalog: 16000-1-AP, Proteintech, China), anti-Caspase 3(1: 500 dilution; Catalog: 27525, SAB, China), anti-Caspase 7(1:1000 dilution; Catalog: 52844, SAB, China), anti-Caspase 9(1:500 dilution; Catalog: 40675, SAB, China), anti-Cleaved Caspase 3(1:500 dilution; Catalog: 40500, SAB, China), anti-Cleaved Caspase 9(1:500 dilution; Catalog: 40504, SAB, China) anti-PARP1(1:500 dilution; Catalog: 38592, SAB, China), anti-Cleaved PARP(1:1000 dilution; Catalog: 48805, SAB, China), and then with a secondary antibody (1:2000 dilution; Catalog: 7074, Cell Signaling Technology, US) at room temperature for 60 min. After washing the membranes three times, a chemiluminescence kit (Catalog: ECL-001, ZhuangzhiBio, China) was used to detect the specific protein bands.

### Cell viability analysis

The viability of FGL1_NC and FGL1_KD Lewis lung carcinoma cells was determined at 24, 48, 72, 96, and 120 h by the CCK-8 assay (Catalog: ST1006.05, Saint Bio, China). Briefly, 2,000 cells in 200 μL DMEM were mixed with 20 μL CCK-8 agent at each time point, and incubated for 1–4 h at 37 °C. Cell viability was determined according to the optical density value determined at 490 nm using a microplate reader.

### Cell proliferation analysis

FGL1_NC and FGL1_KD Lewis lung carcinoma cells were collected, resuspended in 1 mL RPMI 1640, and cell density was measured using an electronic cell counter (Olympus, Tokyo, Japan). Briefly, 2,000 cells were seeded in 150 μL RPMI 1640 in an E-Plate (Agilent Technologies, Santa Clara, CA, USA). Then, the plate was placed in a real-time cell analyzer (Agilent Technologies) and the cell proliferation was evaluated in following 96 h.

### LUAD experimental mouse model

Male mice (BALB/cJGpt-Foxn1^nu^/Gpt; 6 weeks old; 20–25 g) were purchased from GemPharmatech (Beijing, China), and were housed with free access to sterilized food and autoclaved water. The mice were randomly separated in two groups (*n* = 5 in each group) and injected subcutaneously (above of hind legs) with FGL1_NC and FGL1_KD Lewis lung carcinoma cells (2×10^6^ cells/animal). From day 7 post-injection forward, the volume of the tumors was measured daily based on the two largest perpendicular dimensions, until the volume of the largest tumor was approximately 1,000 mm^3^ and the animals were euthanized. The tumor volume was calculated as follows: tumor volume (mm^3^) = (tumor length [mm] × square of tumor width [mm]^2^)/2. All animal experiments were conducted according to the guidelines and approved by Ethics Committee of the Air Force Medical University. (Ethics Approval number: 20220666).

### Gene expression analysis

The levels of *YY1* and *FGL1* in PC9 cells were determined by real-time quantitative polymerase chain reaction (RT-qPCR) using the 2^−ΔΔCt^ method with *ACTB* as internal control. Briefly, total RNA was extracted using miRNeasy mini kit (Qiagen, Hilden, Germany) and gene expression was evaluate using TaqManfi Gene Expression Assays (Thermo Fisher Scientific), according to the manufacturers’ protocols.

### Chromatin immunoprecipitation (ChIP)-qPCR

ChIP was performed using ChIP kit (Catalog: Bes5001, BersinBio, China) with a specific FGL1 immunoprecipitation antibody (ab170922; Abcam, Cambridge, United Kingdom) or normal rabbit IgG antibody. Next, qPCR was performed to amplify and quantify the immunoprecipitated DNA. All qPCR analyses were completed using CFX Manager Software 1.6 (Bio-Rad, Hercules, CA, USA) with the 2^−ΔΔCt^ method, GADPH served as the control.

### Luciferase reporter assay

Four YY1–FGL1 binding sites were mutated in the *FGL1* promoter. FGL1-promoter-WT/Renilla and FGL1-promoter-MT/Renilla Plasmids carrying the wild-type and mutated FGL1 promoters were cotransfected with the PEGFP-N1-YY1 Plasmids into 293T cells by Lipsome 2000 (Catalog: 11668019, Thermo Invitrogen). Then, *FGL1* promoter activity was measured after 24 h using a Dual-Glo Luciferase Assay Kit (Beijing Tsingke Biotech Co.).

### Immunoprecipitation and mass spectrometry

Total protein was obtained from PC9 cells using a total protein collection kit (Invent Biotechnologies Inc.). Briefly, SD-002 buffer was used to lyse the cells on ice for 15 min and the solution was then centrifuged at 12,000 rpm for 30 s to remove the debris. The lysates were incubated at 4 °C for 8h with a specific FGL1 immunoprecipitation antibody (ab170922; Abcam) and Protein A/G agarose was then incubated for additional 2 h. The samples were extensively washed with lysis buffer and were analyzed by mass spectrometry (performed by Beijing TsingKe Biotech Co.).

### Statistical analysis

Graphpad Prism 8.2.1 software was used for statistical analysis, statistical data were expressed as mean ± standard deviation (SD). The differences between two groups were evaluated by Student’s t test. For data without equal SD, Mann–Whitney test was used to compare the ranks. *P* ≤ 0.05 was considered statistically significant (*P < 0.05; **P < 0.01; ***P < 0.001; ****P < 0.0001).

## Results

### *FGL1* expression in LUAD is positively associated with *PD-L1* (*CD274*) but not *PD1* levels

**Figure 1:**
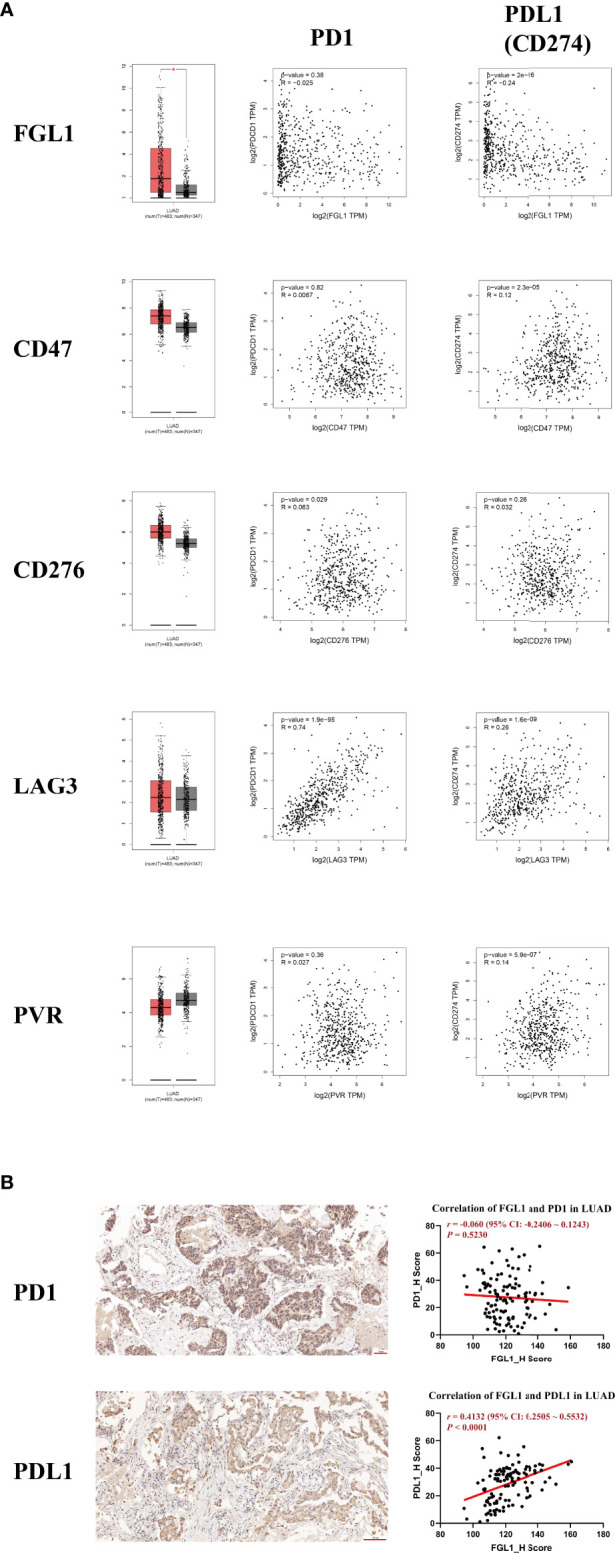
The expression of FGL1 in LUAD is positively associated with PD-L1 (CD274) but not with PD1. **(A)** Five of the most studied immune checkpoint genes (*FGL1, CD47, CD276, LAG3, PVR*) with the greatest potential for clinical transformation were included and analyzed using GEPIA. **(B)** The representative figures of PD1 and PD-L1 staining by IHC in LUAD are presented with correlation analysis. FGL1 is positively associated with the expression of PD-L1 (*r* = 0.4132, *P* < 0.0001), but there is no correlation of FGL1 with PD1. LUAD, lung adenocarcinoma; IHC, immunohistochemistry.

Five immune checkpoint genes (*FGL1*, *CD47*, *CD276*, *LAG3*, and *PVR*) previously described as potential oncogenes were evaluated further based on data from the GEPIA database. In particular, were evaluated their differential expression in LUAD and their correlation with *PD1* and *PD-L1* (**Figure 1A**). Interestingly, only FGL1 was found to be significantly differentially expressed in LUAD (*P* < 0.05). Moreover, the correlation between *LAG3* and *PD1* was the most significant (*r* = 0.74, *P* < 1×10^−4^), whereas *CD276* weakly correlated with *PD1* levels (*r* = 0.063, *P* = 0.029). Similarly, a weak but statistically significant correlation (*|r*| < 0.3, *P* < 1×10^−4^) was observed between *FGL1*, *CD47*, *LAG3*, and *PVR* with *PD-L1*. Only *FGL1* was negatively associated with the expression of *PD-L1* (*r* = −0.24, *P* < 1×10^−4^), whereas others were positively associated with this marker. Considering the current research status, we choose FGL1 as our target. To further explore these molecular relationships, 140 LUAD intratumoral and paired peritumoral primary samples were evaluated with focus on FGL1 expression. Overall, FGL1 was found to be positively associated with the expression of PD-L1 (*r* = 0.4132, *P* < 1×10^−4^), but no correlation between FGL1 with PD1 was observed (**Figure 1B**). Thus, we considered that in addition to serve as an independent immune checkpoint gene, FGL1 may also be coexpressed with PD-L1 and further play a regulatory role in tumor immunity.

### FGL1 is positively associated with CD3D expression but negatively associated with FOXP3, S100A9, and TPSB2

**Figure 2:**
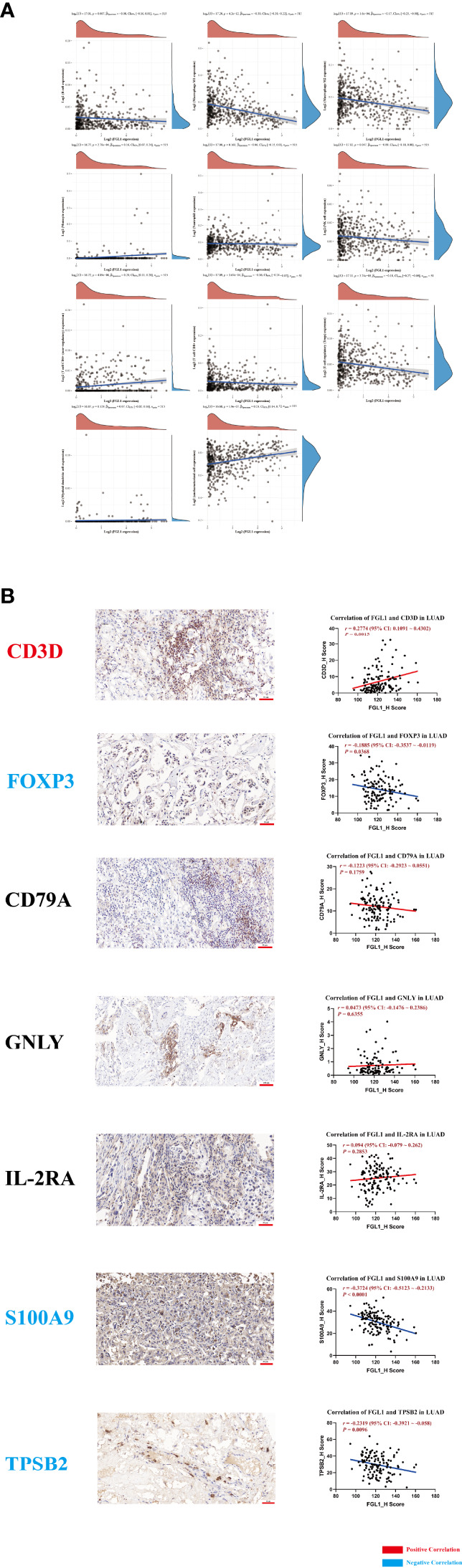
FGL1 is positively associated with CD3D expression and negatively associated with FOXP3, S100A9, and TPSB2. **(A)** Immune infiltration analysis was conducted using the ACLBI database. **(B)** The representative figures of CD3D, FOXP3, CD79A, GNLY, IL-2RA, S100A9 and TPSB2 staining in LUAD are presented; the correlation between FGL1 and immune markers was assessed. FGL1 is positively correlated with CD3D (T cell marker) expression (*r* = 0.2774, *P* = 0.0015) but negatively associated with FOXP3 (Treg marker) expression (*r* = -0.1885, *P* = 0.0368), S100A9 (neutrophil marker) expression (*r* = -0.3724, *P* < 0.0001), and TPSB2 (mastocyte marker) expression (*r* = -0.2319, *P* = 0.0096).

A potential immune regulatory role of FGL1 could have a detrimental impact the outcome of PD-L1-related immunotherapy in LUAD. Therefore, we performed immune infiltration analysis to explore the specific role of FGL1 in LUAD. *FGL1* was found to be negatively associated with macrophage M1 (*r* = −0.30, *P* = 4.2×10^−12^), macrophage M2 (*r* = −0.17, *P* = 1.6×10^−4^), natural killer cell (*r* = −0.09, *P* = 0.047), CD8^+^ T cell (*r* = −0.16, *P* = 3.67×10^−4^), and T regulatory cell (*r* = −0.18, *P* = 3.74×10^−5^) expression, whereas was positively associated with monocyte (*r* = 0.16, *P* = 2.78×10^−4^) and CD4^+^ T cell (*r* = 0.20, *P* = 4.89×10^−6^) expression in LUAD tissues (**Figure 2A**). Further analysis of primary LUAD tissue samples further showed that FGL1 was positively correlated with CD3D (T cell marker) levels (*r* = 0.2774, *P* = 1.5×10^−3^), but negatively associated with FOXP3 (T regulatory cell marker) (*r* = −0.1885, *P* = 0.0368), S100A9 (neutrophil marker) (*r* = −0.3724, *P* < 1×10^−4^), and TPSB2 (mastocyte marker) (*r* = −0.2319, *P* = 9.6×10^−3^) levels (**Figure 2B**). Taken together, these results suggested that FGL1 may have an immune regulatory effect in LUAD, especially for T cells.

### FGL1 promotes the secretion of IL-2 by Jurkat cells and induces their apoptosis

**Figure 3:**
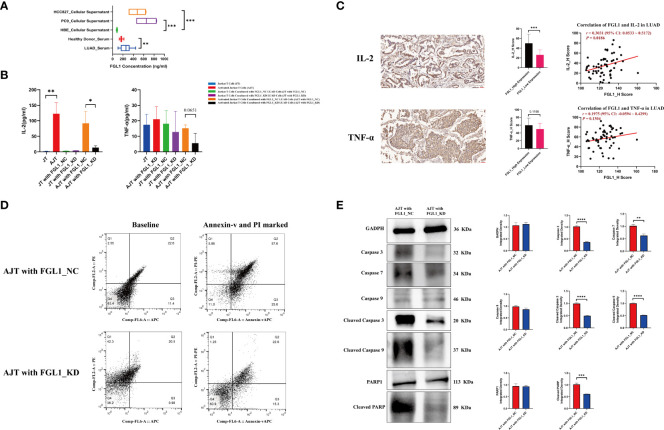
FGL1 promotes the secretion of IL-2 from Jurkat T cells, simultaneously inducing the apoptosis of Jurkat T cells. **(A)** The concentration of FGL1 in serum and cellular supernatant samples detected using ELISA. **(B)** The contents of IL-2 and TNF-α detected by ELISA after co-culture. **(C)** The protein levels of IL-2 and TNF-α were evaluated using IHC in 60 LUAD samples. **(D)** The knockdown of FGL1 in PC9 cells effectively inhibits the apoptosis of activated Jurkat T cells. **(E)** The results of a western blot show the differences in apoptosis markers between the “activated Jurkat T cells cocultured with FGL1_NC PC9 cells” group and the “activated Jurkat T cells cocultured with FGL1_KD PC9 cells” group; caspase 3, caspase 7, cleaved caspase 3, cleaved caspase 9 and cleaved PARP, all decreased in the “activated Jurkat T cells cocultured with FGL1_KD PC9 cells” group. LUAD, lung adenocarcinoma; IHC, immunohistochemistry. *, P < 0.05; **, P < 0.01; ***, P < 0.001; ****, P < 0.0001.

Next, the levels of FGL1 were evaluated by ELISA in 12 healthy donor serum samples and 32 LUAD serum samples, as well as in six HBE, four PC9 and four HCC827 supernatant samples. The mean concentrations of FGL1 were 172.76 ng/mL in healthy donor serum samples, 255.57 ng/mL in LUAD serum samples, and 83.70 ng/mL in HBE samples, whereas they were 654.18 and 477.67 ng/mL in the PC9 and HCC827 samples, respectively (**Figure 3A**). Previous reports demonstrated that FGL1 is a ligand of LAG3 and that their interaction can deregulate the immune balance (8). Thus, along with our finding, FGL1 may be secreted into the serum and bind to LAG3 to exert its immune regulatory effects.

The ability of FGL1 to regulate the activity of T cells was further evaluated *in vitro* using Jurkat cells activated by phytohemagglutinin. While the presence of phytohemagglutinin significantly promoted the secretion of IL-2 (*P* < 0.01) as compared with non-activated cells, coculture with PC9 cells lacking FGL1 led to a significantly reduced secretion of IL-2 by Jurkat cells than in cells cocultured with control PC9 cells (*P* < 0.05) (**Figure 3B**). Similar results were observed for TNF-α, with TNF-α content in the coculture supernatant being lower in the presence of FGL1 KD cells compared with control cells (*P* = 0.0651) (**Figure 3B**). IFN-γ was not detectable in the culture supernatant by the method used; moreover, the protein levels of IL-2 and TNF-α were evaluated using IHC in 60 LUAD samples. Quartiles were considered cutoff values, H scores less than 25% were considered low expression, and those more than 75% were considered high expression. As compared to that in the FGL1-low expression group, the results of IHC indicated that the FGL1 high-expression group had higher IL-2 (*P* < 0.001), and the correlation analysis also confirmed that FGL1 is positively associated with IL-2 (*r* = 0.3031, *P* = 0.0186) but not for TNF-α (*r* = 0.1975, *P* = 0.1304) (**Figure 3C**). Further analysis of these cells by flow cytometry showed that the presence of PC9 cells lacking FGL1 effectively prevented the apoptosis of activated Jurkat cells (**Figure 3D**) (22.6% *vs*. 57.6%), with reduced levels of various apoptosis markers, including caspase 3, caspase 7, cleaved caspase 3, cleaved caspase 9, and cleaved PARP (**Figure 3E**). These results indicate that FGL1 plays an important immune regulatory effect in T cells in LUAD.

### FGL1 is positively associated with the proliferation of LUAD cells

**Figure 4:**
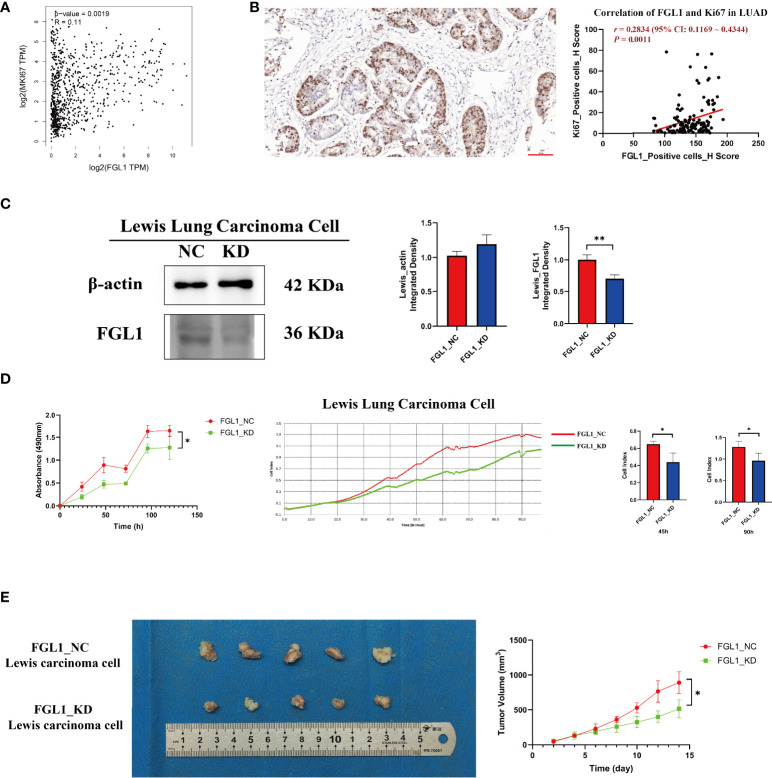
FGL1 is positively associated with Ki67 expression and Lewis lung carcinoma cell proliferation. **(A)** Positive mRNA correlation between FGL1 and MKI67, performed in GEPIA (*r* = 0.11, *P* = 0.0019). **(B)** Positive protein correlation between FGL1 and Ki67, found using LUAD tissue ChIPs (*r* = 0.2834, *P* = 0.0011). **(C)** FGL1 was knocked down in Lewis lung carcinoma cells, shown by Western blot. **(D)** CCK-8 and real-time cell analyzer indicated that the downregulation of FGL1 inhibited Lewis lung carcinoma cell proliferation (*P* < 0.05). **(E)** The representative figure of a subcutaneous tumor bearing model was presented. A tumor model in mice also proved that the knockdown of FGL1 significantly inhibited tumor growth (*P* < 0.05). *, P < 0.05; **, P < 0.01.

Previously, we demonstrated that FGL1 expression promoted the proliferation of PC9 and HCC827 cells (18). In agreement with these results, additional analysis of GEPIA data confirmed that *FGL1* was positively associated with the expression of MKI67 (a proliferation marker) at the mRNA (*r* = 0.11, *P* = 1.9×10^−3^) (**Figure 4A**) and protein levels (*r* = 0.2834, *P* = 1.1×10^−3^) in LUAD (**Figure 4B**). Further experiments confirmed that FGL1 KD (**Figure 4C**) prevented the proliferation of Lewis lung carcinoma cells both *in vitro* (**Figure 4D**) and *in vivo* (*P* < 0.05) (**Figure 4E**).

### YY1 is upstream to FGL1 and participates in its transcriptional regulation

**Figure 5:**
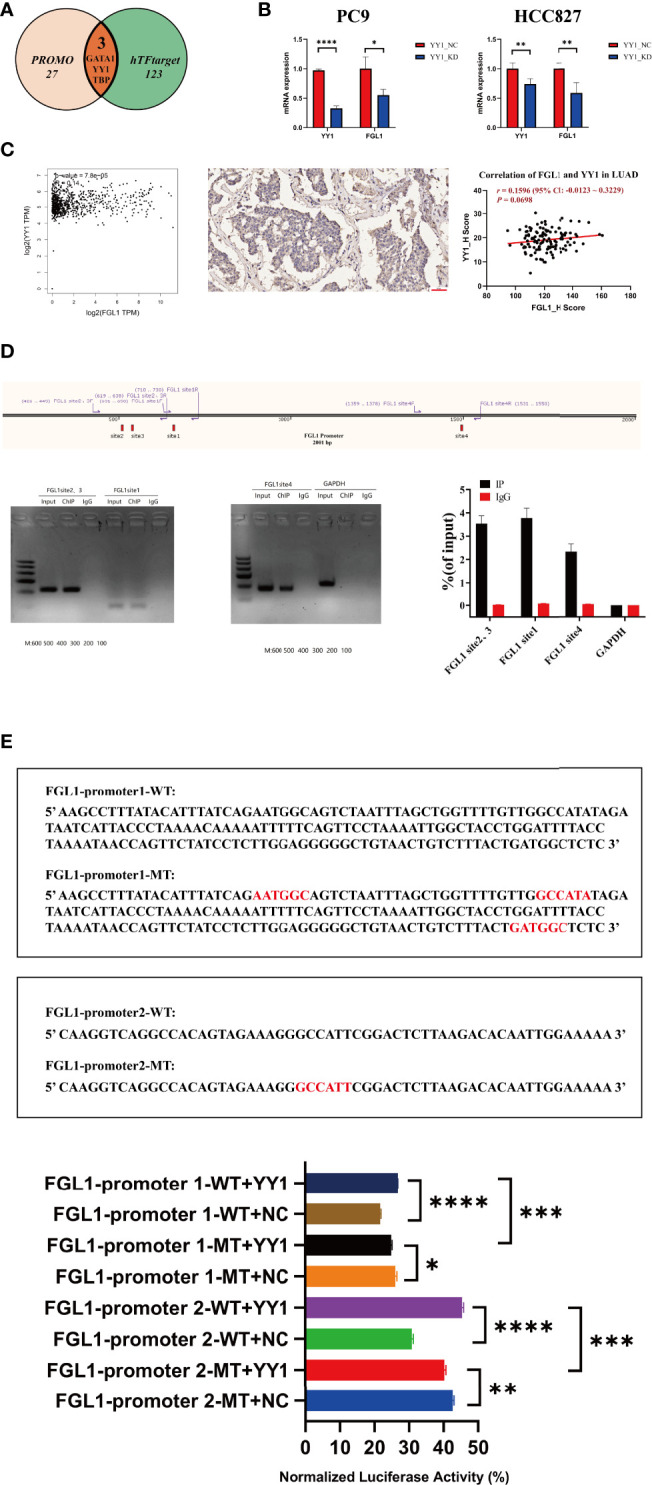
YY1 is an upstream molecule of FGL1 and participates in the transcriptional regulation of FGL1. **(A)** Transcription factor of FGL1 was predicted in PROMO and hTFtarget. **(B)** The level of YY1 mRNA was knocked down by siRNA in PC9 and HCC827 cells (*P* < 0.0001); the mRNA expression of FGL1 also decreased (*P* < 0.05). **(C)** The correlation of YY1 and FGL1 was predicted in GEPIA and validated in tissue ChIP. **(D)** ChIP-qPCR was performed in PC9 cells to further verify the direct binding of YY1 and FGL1. **(E)** Dual luciferase reporter assay was performed in HEK 293T cells, which shows once again that YY1 is the transcription factor of FGL1 and has four binding sites. ChIP, chromatin immunoprecipitation. *, P < 0.05; **, P < 0.01; ***, P < 0.001; ****, P < 0.0001.

The specific underlying mechanisms of FGL1 regulation in LUAD were explored next. PROMO and hTFtarget databases were used to predict potential regulatory transcription factors of *FGL1*, among which were GATA1, YY1, and TBP, which are all found at higher levels in the lung (22–24). As YY1 was found to be more highly expressed in the lung (22) and lung adenocarcinoma (25), it was selected for further analysis (**Figure 5A**). Interestingly, KD of YY1 by siRNA in PC9 cells (*P* < 1×10^−4^) and HCC827 cells (*P* < 0.01) was accompanied by reduced expression of *FGL1* (*P* < 0.05 in PC9, *P* < 0.01 in HCC827) (**Figure 5B**). Indeed, GEPIA data also suggested that *YY1* was positively associated with *FGL1* (*r* = 0.14, *P* = 7.8×10^−5^), a relationship that was also confirmed in primary tissue LUAD samples (*r* = 0.1596, *P* = 0.0698) (**Figure 5C**). ChIP-qPCR analysis of PC9 cells revealed four binding sites between YY1 and FGL1 (**Figure 5D**), among which the sites 1, 2, and 3 were close, whereas site 4 was relatively far away. To confirm the regulatory relationship between YY1 and FGL1, dual luciferase reporter assay was performed in 293T cells. Noteworthily, concomitant mutation of sites 1, 2, and 3 in the *FGL1* promoter led to reduced *FGL1* expression (**Figure 5E**), once again proving that YY1 is a transcription factor of *FGL1*.

### MYH9 is the downstream effector molecule of FGL1 in LUAD

**Figure 6:**
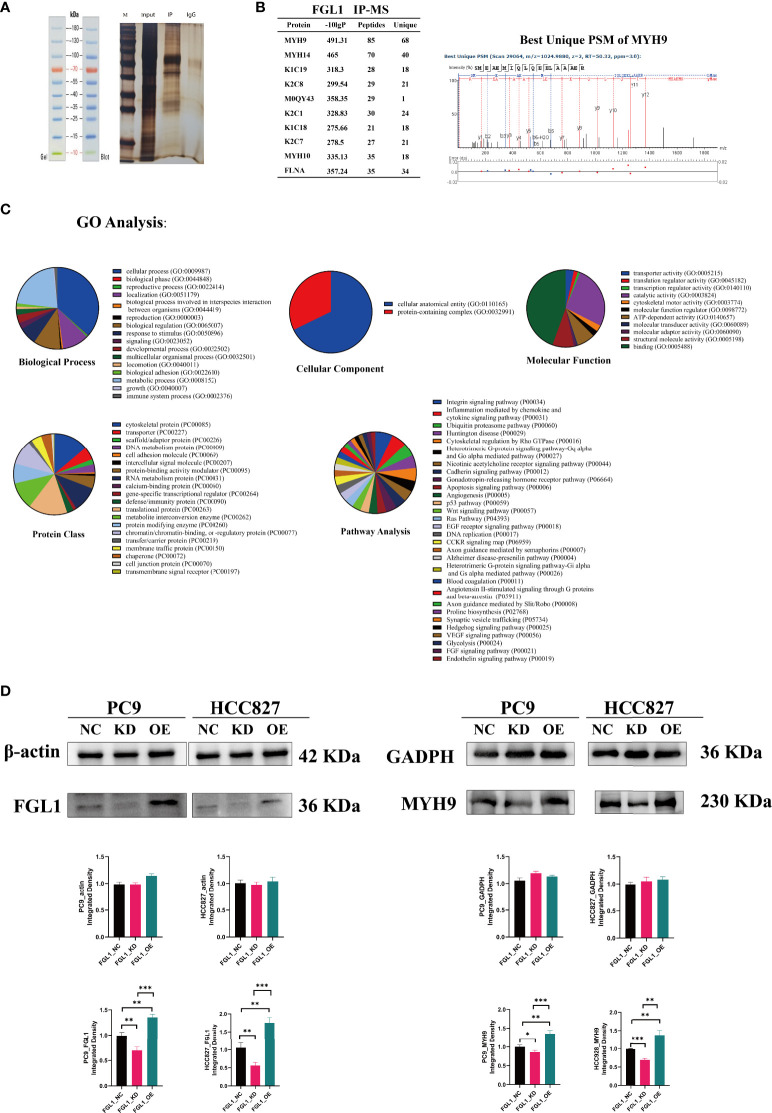
MYH9 is a downstream molecule of FGL1. **(A)** IP was used to adsorb and precipitate related proteins that FGL1 might bind to. **(B)** MS was performed and the top 10 FGL1-combined proteins were listed—MYH9 was the most likely protein to bind to FGL1. **(C)** GO analysis of the results of MS with five subclasses, including biological process, cellular component, molecular function, protein class, and pathway analysis. **(D)** Validation by western blot supports that MYH9 is the main downstream molecule of FGL1. IP, immunoprecipitation; MS, Mass spectrometry. *, P < 0.05; **, P < 0.01; ***, P < 0.001.

Next, to explore possible direct partners of FGL1 in LUAD cells, FGL1-related proteins were collected by immunoprecipitation (**Figure 6A**) and were then identify by mass spectrometry. Among the top 10 FGL1-associated proteins, MYH9 was the most likely protein to bind to FGL1 and be an effector regulatory partner (**Figure 6B**). Western blot validation also supported that MYH9 is the main downstream molecule of FGL1, with the downregulation and upregulation of FGL1 expression inducing reduced and increased expression of MYH9, respectively, in PC9 and HCC827 cells (**Figure 6D**).

Gene ontology analysis of FGL1 concerning five subclasses further showed that: concerning biological processes analysis, FGL1 was mainly involved in cellular and metabolic processes; regarding cellular components, FGL1 was mainly known to interact with cellular anatomical proteins; concerning the molecular function, FGL1 and its downstream proteins were mainly involved in cell binding; regarding protein classes, cytoskeletal proteins and translational proteins were the mainly affected proteins by FGL1 activity; and that concerning pathways, FGL1 mainly affected the activity of integrin signaling pathway and inflammation mediated by chemokine and cytokine signaling pathways (**Figure 6C**), which could be associated with the regulation of IL-2 secretion herein described (**Figure 3**).

Taken together, these findings suggest that FGL1 may play a role in the secretion of inflammation-related chemokines, and mainly interacts with a cytoskeletal protein to further regulate the cellular process and cell proliferation. In agreement with these hypotheses, our previous report also indicated that FGL1 downregulation can induce the arrest of LUAD cells in the G0/G1 phase (18).

## Discussion

The high incidence and mortality of lung cancer (1) are a clear indication that we are still in a long way from finding a cure for this malignancy. Indeed, new immunotherapies that can induce enhanced anticancer responses with fewer immune-related adverse effects are urgently need. The data collected in the present study suggest that FGL1 may be a valuable target candidate for the development of such novel treatment strategies.

Herein, we demonstrated that FGL1 can promote the secretion of important immune regulatory cytokines (IL-2) by T cells, but it also induces the apoptosis of these immune cells. Noteworthy, FGL1 is positively associated with CD4^+^ T cell levels within the tumor, while it can also support the expression of CD3D and prevent that of FOXP3; hence, FGL1 may support T cell tumor infiltration and function in the cancer microenvironment. Moreover, FGL1 was found to be positively correlated with PD-L1 expression, which is known to be an important checkpoint immune protein in tumors (26, 27). Importantly, herein we demonstrate for the first time that FGL1 can prevent immune regulation and induce apoptosis in LUAD, which is consistent with the results of other reports that demonstrate the inhibitory immune regulation effect of FGL1 in other tumors (8, 17, 28). Hence, the negative immune regulatory effect of FGL1 than its positive regulatory effect, which overall results on the inhibition of the immune response in LUAD.

Gene ontology analysis suggested that FGL1 could interact with cytoskeletal proteins and be involved in cellular processes to regulate LUAD proliferation, and that FGL1 could also regulate inflammation *via* chemokine and cytokine signaling pathways, possibly through IL-2 secretion by T cells based on our findings. Indeed, mechanistic assessment revealed that the YY1–FGL1–MYH9 axis plays a pivotal role in LUAD immunity and proliferation both *in vitro* and *in vivo*. We demonstrated that YY1 can bind to *FGL1* promoter at four different sites, which will consequently support FGL1 expression. Then, FGL1 was found to be positively associated with the expression of Ki67 and might cooperate to promote the proliferation of LUAD cells.

In summary, FGL1 plays an immunological and proliferative role in LUAD, by promoting the secretion of IL-2 by T cells and inducing the apoptosis of these cells. Furthermore, the YY1–FGL1–MYH9 axis plays an important role in cellular process regulation, as well as on tumor proliferation and immunity, *via* chemokine and cytokine pro-inflammatory signals. Finally, due to the strong double regulation of FGL1 in LUAD, FGL1 may represent a relevant therapeutic target for cancer immunotherapy, especially for LUAD.

## Data availability statement

The datasets presented in this study can be found in online repositories. The names of the repository/repositories and accession number(s) can be found in the article/supplementary material.

## Ethics statement

The studies involving human participants were reviewed and approved by Ethics Committee of the Air Force Medical University (No. 202003-018). The patients/participants provided their written informed consent to participate in this study. The animal study was reviewed and approved by Ethics Committee of the Air Force Medical University. (Ethics Approval number: 20220666).

## Author contributions

X-YT, Y-LX, Y-BZ, and JY collected the related papers and drafted the manuscript, A-PS, K-FZ, Y-JL, and CS participated in the design of the review, J-BZ, NM, and TJ initiated the study and revised and finalized the manuscript. All authors read and approved the final manuscript.

## Funding

This work was supported by the National Natural Science Foundation of China (No. 82002421; No. 81001041).

## Acknowledgments

All those involved and their families deserve our thanks, we are very grateful for National Natural Science Foundation of China funding us.

## Conflict of interest

The authors declare that the research was conducted in the absence of any commercial or financial relationships that could be construed as a potential conflict of interest.

## Publisher’s note

All claims expressed in this article are solely those of the authors and do not necessarily represent those of their affiliated organizations, or those of the publisher, the editors and the reviewers. Any product that may be evaluated in this article, or claim that may be made by its manufacturer, is not guaranteed or endorsed by the publisher.

## Abbreviations

LUAD, lung adenocarcinoma; NSCLC, non-small cell lung cancer; irAEs, immune-related adverse events; FGL1, Fibrinogen-like protein 1; LAG3, Lymphocyte-activation gene 3; YY1, YIN-YANG-1; MYH9, Myosin Heavy Chain 9; DMEM, Dulbecco’s Modified Eagle Medium; ELISA, enzyme-linked immunosorbent assay; FCS, fetal calf serum; GEPIA, Gene Expression Profiling Interactive Analysis; GTEx, Genotypic-Tissue Expression; TCGA, The Cancer Genome Atlas; ACLBI, Assistant for Clinical Bioinformatics; hTFtarget, Human Transcription Factor Targets; IL, interleukin; INF, interferon; KD, knocked down; NC, negative control; PCR, Polymerase Chain Reaction; ChIP-qPCR, Chromatin immunoprecipitation-quantitative polymerase chain reaction; IP, immunoprecipitation; MS, Mass Spectrometry; SD, standard deviation.
